# Introduction of an undergraduate interprofessional simulation based skills training program in obstetrics and gynaecology in India

**DOI:** 10.1186/s41077-019-0096-7

**Published:** 2019-04-18

**Authors:** Suhas Gorantla, Utkarsh Bansal, Jai Vir Singh, Akhilesh Dutta Dwivedi, Atul Malhotra, Arunaz Kumar

**Affiliations:** 1 Monash University, Melbourne, Australia; 2 Hind Institute of Medical Sciences, Lucknow, Uttar Pradesh India; 3 Department of Paediatrics, Monash University, Melbourne, Australia; 4 Department of Obstetrics and Gynaecology, Monash University, Clayton, Melbourne, VIC Australia

**Keywords:** Pre-registration, Labour, Speculum, Vaginal, Examination, Post-partum haemorrhage

## Abstract

Interprofessional simulation based education (SBE) improves core clinical skills and team training in obstetrics and gynaecology. In this innovative study, the introduction of an undergraduate interprofessional SBE program for teaching obstetrics and gynaecology skills in India was evaluated. The study attempted to evaluate the feasibility and benefit of the interprofessional skills training workshop in obstetrics and gynaecology, which was introduced for medical and midwifery students in a secondary level hospital in India. The program focuses on improving “hands-on” clinical skills and can be explained by the “skills acquisition theory”. Using a survey, participants rated relevance, pitch and confidence (on a 5-point Likert scale) and described the contextualisation and teaching of core clinical skills through the workshop using free-text. Descriptive analysis of quantitative Likert scale responses and thematic analysis of the free-text data was conducted and themes identified. Ninety-five medical and midwifery students attended the inaugural workshop, in a low-resource setting. The clinical experience in obstetrics and gynaecology across both groups was minimal, neither were they exposed to any prior SBE. Both health professional groups found the workshop useful, relevant and improved their confidence in performing vaginal examination and births. The key theme, which emerged from qualitative analysis, was “getting hands-on” experience. Other themes included learning by simulation without clinical time constraints, retaining the ability to make mistakes, bridging theory to practice, valuing interprofessional experience and ensuring equal learning opportunities for all participating professional groups. The advantages of interprofessional SBE, for medical and midwifery students, are reproducible in a low-resource setting, and may be be helpful for learning intimate clinical examination, obstetric procedures and team training.

## Background

Acquisition of clinical skills is important for undergraduate medical students during their training in obstetrics and gynaecology. This experience is mostly acquired from placements on birth units and clinics where students learn about performing obstetric examination and births. Similar standards are also expected for midwifery students, where they are encouraged to be directly involved in the care of pregnant women [1] during placements. When these students eventually become doctors or midwives, they are generally expected to perform internal examinations for women independently. However, due to the lack of opportunity to practice skills in a busy clinical environment, students may often lack confidence and may not have acquired the necessary skills during their training [2].

Simulation based education (SBE) provides students with “scaffold learning” to develop skills and competence in examining patients [3]. It bridges the gap between “theory to practice”, as knowledge learnt in a classroom can be practiced on simulators prior to real patients. Studies have revealed that SBE can increase medical students’ perceived comprehension of common gynaecology [4, 5] and obstetric procedures, improve their confidence with vaginal births, and boost their accuracy in cervical examinations [6]. Similar findings have been reported when simulation was used in educating midwifery students, where it can assist in the development of integrated and global clinical skills [7].

Obstetrics is a field that relies heavily on teamwork and communication between multiple disciplines, where nurses, midwives, obstetricians and paediatricians must work together under intense time pressure to make pivotal decisions. Team training in clinical teams has proven to improve performance under adversity and stress, promote cooperation and reduce errors arising from miscommunication [8]. However, the benefits of starting interprofessional training early in their training amongst undergraduate students of both faculties have not been researched sufficiently. Complications in timetable rostering and differences in tertiary education providers for each faculty have made undergraduate interprofessional training difficult to implement. If interprofessional education (IPE) can be implemented, it can increase students’ exposure to other professions and allow them to develop unprejudiced impressions of other students before they graduate and develop their professional identities in their respective workplace [9].

In addition, in middle-income countries, like China and India, students often find it difficult to gain clinical exposure, as patients may refuse to have medical students involved in their care. This may be due to issues like increasing mistrust between the medical profession and patients, where patients have even declined to be operated on by resident surgeons [10]. Coupled with a relatively limited “hands-on” time in each rotation, students may struggle to acquire adequate clinical experience to perform procedures and examinations confidently. However, in spite of simulation demonstrating evidence of learning in a low-risk environment to practice and master skills, a search into the literature revealed that it has not been trialled extensively in these middle-income countries. This might be due to uncertainty over its cost-effectiveness and a lack of advocacy in clinical institutions [10].

Our study is set up in India, where neither SBE nor IPE is common. Recently, we introduced IPE for medical and midwifery staff in obstetric and neonatal emergency training [11]. In the present study, we introduced a program called Women’s’ Health Interprofessional Learning by Simulation (WHIPLS) in the Indian setting. This program has already been shown to be beneficial in a high-resource setting [1, 9, 12] and is well integrated into both medical and midwifery curriculum in an Australian university [12]. This study explores the feasibility of introducing WHIPLS in a low-resource setting. It aims to evaluate the effectiveness of an education program using simulation in teaching core clinical skills to medical and midwifery students by assessing students’ perceptions of the program. Additionally, by having medical and midwifery students learn together, it explores the benefits of early interprofessional training in obstetrics and gynaecology.

## Methods

### Setting and design

This study was conducted at Hind Institute of Medical Sciences, India using an exploratory research design. Hind Institute offers undergraduate medical and nursing education along with a secondary level hospital, co-located in the same campus in Barabanki district (near Lucknow), Uttar Pradesh, India. The obstetrics and gynaecology department of the hospital looks after women with gynaecological conditions, pregnant women delivering infants more than 34 weeks gestation, and has outpatients, inpatient wards, delivery rooms and operating theatres.

### Participants

Fourth-year medical students who were embarking on their obstetrics and gynaecology rotation, and midwifery students who were in their final year of nursing training were invited to participate in the study. The university enrols approximately 100 medical and 240 combined nursing and midwifery students annually. At the time of attendance, the medical students had received relevant didactic lectures but had not been exposed to any clinical experience in this field. The midwifery students were enrolled in a combined 4-year nursing and midwifery course, and had received some hands-on clinical experience in the birth unit.

### Workshop

The WHIPLS program was being introduced in India for the first time. The program included a 3-h clinical skills workshop, which was complemented by a blend of online-lectures, pre-reading material and videos provided to the participants beforehand [1]. The lectures covered essential theoretical information necessary for safe performance, and described the relevance of the clinical skills. These clinical skills were identified as an essential component of core curriculum for both professions. The program content was matched to both the medical and midwifery curricula and was designed and approved by both curriculum leaders. The learning objectives were relevant to both professional groups, which included safe handling of instruments, ensuring patient safety and comfort during clinical examination, conducting clinical procedures of low complexity as a team of medical and midwifery students and identification of complications.

The theoretical basis of the WHIPLS program relates to the “skills acquisition model” explained by Fitts and Posner in 1967 [13]. According to the model, there are three stages of skills acquisition [14], described as cognitive, where the learner learns to perform the procedure using small steps or manipulate the instruments (e.g. the vaginal speculums), the integrative or associative stage (where the learner associates the knowledge with the skill and is able to “make sense of” applying that skill (e.g. inserting the speculum in a simulator, engaging with the simulator and performing the task in the clinical context). In this stage, the learner may still be thinking about how to perform the skill, but is slightly more fluent in performance. And finally the autonomous stage, where the learner does not need to actively think or work out the steps of the procedure (e.g. inserting the speculum with some degree of automation, but is able to think about other things like the patient’s response to the procedure and communication with the patient).

A skills station circuit was formed, where groups of 6–8 students practiced for an hour at each station, which was led by a facilitator. The facilitator team comprised of local midwifery and medical staff with extensive experience in the fields of obstetrics and gynaecology and were also involved in teaching medical and midwifery students.

The stations consisted of the following skills training:

1. Speculum, bimanual examination and performing a cervical screen test
2. Vaginal examination and assessment in labour
3. Conducting a normal vaginal birth with estimation of blood loss

The simulators used were Zoe Gynaecologic simulator (Abacus dx, Meadowbrook, Australia) and PROMPT flex birthing simulator (Limbs and Things, Bristol, UK). At each station, the facilitators initially demonstrated the procedure on task trainers to students who were then supervised performing the procedure independently. Individual and group feedback was given to the students at each skill-station. Procedural skills were taught in the context of clinical scenarios where relevant and clinical cases were discussed. Although students from both professions performed the skills individually at a given time, the program encouraged peer interaction between the two groups, sharing knowledge and supporting peer learning.

### Surveys

Students were asked to fill a 15-min paper-based pre-survey (16 items) and another 15-min post-workshop evaluation survey (10 items on a Likert’s scale and three questions with free-text responses). In the pre-workshop survey, the details of demographics (age, gender, year of training) of the participants were obtained through the pre-workshop survey and about their level of training, number of vaginal births, exposure to obstetrics and gynaecology examinations, and cervical screen test they had witnessed, assisted in or performed. They rated their level of confidence in conducting these examinations themselves and whether they had heard or participated in any interprofessional activity before. Both surveys have been used in the original publication describing the WHIPLS program and were modified slightly to suit the setting [1].

Upon the completion of the workshop, students were asked to rate the relevance, pitch, explanation of each of the three stations and their confidence in performing the skill independently. These were rated on a 5-point Likert scale (1, strongly disagree; 5, strongly agree). In the section on free-text responses, students were asked to share their thoughts about learning core obstetrics and gynaecology skills in a simulation workshop as opposed to learning during a clinical session or a tutorial; if and how, the workshop was/was not relevant to their clinical experience. They were asked to comment about the value of it being an interprofessional activity. All text data were transcribed verbatim.

### Analysis

Demographic and other numerical data were analysed using descriptive and interpretative statistics (GraphPad Prism, v7, San Diego, CA). *t* tests were performed to compare data between the groups. A *p* value of less than 0.05 was considered significant. Responses to all open-ended questions were entered and qualitative data were thematically analysed [15]. To analyse these responses, a coding framework was developed to identify recurring themes that accurately reflected the students’ perceptions of the workshop. Qualitative analysis was performed by AK (who delivered the teaching and had developed the WHIPLS workshop) and SG (who was an uninitiated medical student), thus, using the combination of the emic (insider involved the intervention) and etic (outsider who was not aware of the details of the intervention) approach. SG and AK independently went through the transcripts and conducted the content analysis. Any discrepancies were negotiated upon, and final themes attributed after discussion.

### Ethics approval

The study was approved by the Institutional Review Board of the Hind Institute of Medical Sciences and assessed as a quality improvement activity.

## Results

**Fig. 1:**
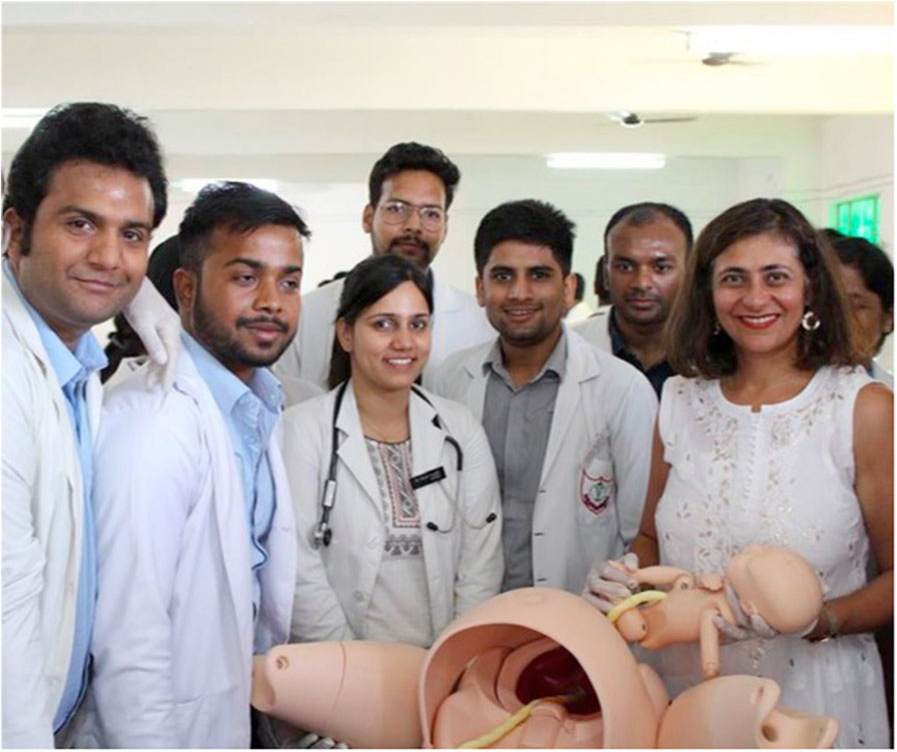
Representative photograph of an obstetrics skills station (conducting normal vaginal birth)

A total of 67 medical students and 28 midwifery students attended the introductory workshop (Fig. 1). The medical students had completed 1 year of clinical rotations prior to the program but had no significant experience in women’s health, while the midwifery students had 1 year of experience in obstetrics and gynaecology. Out of the 95 students who took part in the workshop, 85 complete sets of pre- and post-workshop evaluation forms were collected. Incomplete sets were excluded from this study; hence, a response rate of 89.5% was achieved for the workshop evaluation.

### Pre-workshop survey

Participant data were split into two groups depending on whether they were medical or midwifery students, and the characteristics of each group were compared. Table 1 details the professional characteristics of the participants. The student groups were similarly aged with a median age of 22 years for medical students and 23 years for midwifery students. Midwifery students however had a significantly lower proportion of male members (10.5%) compared to medical students (52.4%, *p* < 0.05). Additionally, both groups had received similar years of clinical training at the time of attendance of the program. Medical students were in their fourth year of training, which is the penultimate year for medical student training and midwifery students were in their final year of training.

**Table 1:** Professional characteristics and experience of workshop participants

	Medical students (*n* = 62)	Midwifery students (*n* = 23)
Age, years	22 (20–27)	23 (20–27)
Male	32 (52.4)	2 (10.5)*
Year of training	4 (2–4)	4 (1–4)
Interprofessional education (IPE)		
Heard of IPE	6 (9.8)	14 (77.7)*
Previous experience of IPE	1 (1.6)	5 (27.7)*
Vaginal exam		
Supervised practice	0 (0–2)	0 (0–6)
Independent practice	0 (0–1)	0 (0–1)
Births		
Observed	0 (0–2)	1 (0–10)*
Assisted	0 (0–0)	0 (0–10)
Performed	0 (0–0)	0 (0–1)
Speculum exam		
Supervised practice	0 (0–0)	0 (0–10)
Independent practice	0 (0–0)	0 (0–1)
Bimanual palpation		
Supervised practice	0 (0–0)	0 (0–10)
Independent practice	0 (0–0)	0 (0–1)
Cervical smear		
Independent practice	0 (0–0)	0 (0–1)

Data expressed as median (range) or *n* (%). * denotes significant difference between groups

The midwifery students were more aware of interprofessional education than their medical student counterparts with 77.7% of midwifery students having heard about IPE previously and 27.7% having participated in it before, compared to 9.8% and 1.6% of medical students (*p* < 0.05) respectively. Similarly, midwifery students had also observed more births, with an average of one birth observed while medical students had seen none. When it came to performing obstetrics and gynaecology examinations with or without supervision, both groups had very little or negligible experience.

### Post-workshop survey

Table 2 shows the post-workshop feedback scores of the two participant groups rating different aspects of the workshop on a Likert scale out of 5. Overall, the results for the post-workshop survey showed that medical students gave an average score of 4.6 and midwifery students an average score of 4.3 out of 5 for the overall benefit of the workshop. The midwifery students in particular found the pre-reading material useful. Simulation of the obstetric stations yielded similar results, as the average scores given by both groups of participants was greater than 4 out of 5 for the various benchmarks that were assessed for each station. While the feedback was similar for gynaecology station, a significantly lower number of midwifery students found the station relevant to their course and were less inclined towards having more sessions in the future.

**Table 2:** Participant post-workshop feedback (Likert scale) scores (out of 5)

	Medical students (*n* = 62)	Midwifery students (*n* = 23)
Overall benefit	4.6 (0.4)	4.3 (0.7)*
Pre-reading material useful	4.4 (0.7)	4.9 (0.2)*
Obstetric stations		
Relevance	4.8 (0.3)	4.7 (0.4)
Pitch	4.6 (0.5)	4.7 (0.4)
Explanation	4.7 (0.4)	4.9 (0.2)
Confidence	4.6 (0.5)	4.6 (0.4)
More sessions	4.7 (0.4)	4.6 (0.5)
Gynaecology stations		
Relevance	4.8 (0.3)	4.5 (0.5)*
Pitch	4.7 (0.4)	4.6 (0.4)
Explanation	4.8 (0.4)	4.9 (0.2)
Confidence	4.7 (0.5)	4.6 (0.4)
More sessions	4.7 (0.4)	4.3 (0.7)*

Data expressed as mean (SD). * denotes significant difference between groups

### Qualitative analysis

The main theme that appeared throughout the post-workshop survey was that the workshop provided “hands-on” learning compared to traditional teaching in tutorials and lectures. The six themes identified from the text data were getting hands-on practice; learning in the simulation without clinical time constraints; retaining the ability to make mistakes; bridging theory to practice; valuing interprofessional experience; and ensuring equal learning opportunities for all participating professional groups. The themes are described below with examples quoted from the medical (indicated as “med”) and midwifery students (indicated as “mid”).

### Getting “hands-on” practice

Both groups of students got practical experience with conducting examinations during the workshop. They had not been exposed to practicing the skills they had learnt in conventional tutorials and cited a lack of opportunity in clinical workplace. Many students reported becoming confident with the examination skills as a primary benefit from the workshop. With the additional practice, they felt better about remembering the procedures and suggested improved confidence in conducting these procedures on real patients.

…. In comparison to clinical sessions, we were able to practice the use of instruments with more confidence … (med)

… It was really very helpful for us as an undergraduate. Because as an undergraduate, we never get such opportunity … (med)

In clinical experience we are only allowed to see the senior staff doing and leading the procedures. But it was entirely different in the workshop … (med)

Simulation workshop provides hands on experience, which is very helpful later in clinical sessions. (mid)

In clinical experience we were not sure about the correct methods of the procedures but now we are confident enough to perform them in the right way … (mid)

… It actually gives you confidence about handling the real patient & gives an imprint on steps on how to perform it [examination or a birth] during a real delivery. This impression will last way longer than normal tutorial class … (med)

### Learning in simulation without clinical time constraints

Students valued the opportunity to practice on the simulators without clinical time constraints. They could practice at a pace that suited their learning without having to rush over any procedures or examinations. Time pressure in busy clinical environments often prevent students from having the opportunity to learn and practice in a flexible manner. According to students, the workshop was able to circumvent that and allow them to learn in a stepwise manner at a self-managed pace.

… as in the clinical setup we actually don't get much time, and we don’t expend this much of time on a single patient … (med)

My thoughts about learning experience through simulation are positive. In placement I have learned about seeing only and not doing as there may not be enough opportunity … (mid)

Much of the learning has been improved by the workshop. Clinical experience is sometimes hurried and somewhat burdensome but the workshop improved the learning to an excellent level through demonstration and practice. (med)

### Retaining ability to make mistakes

Students appreciated the ability to make mistakes without severe consequences during simulation-based training. Many students reported feeling anxious towards performing clinical examinations in real-life clinical settings for fear of causing distress and “actual harm” to patients. Simulation-based training afforded students the luxury to make mistakes and learn from them, which was recognised as an integral aspect of the learning process.

...as it doesn't involve dealing with concern of patients or actual harm to the patient even when this was my first-time experience … (med)

… this will help for practice on the model before applying it (procedural skill) on a real human being.(mid)

My clinical experience is limited, but I had (to conduct) an independent delivery in a hospital during my clinical posting.(mid)

… It is a good opportunity for us to practice first on dummy then on a normal person. That way we can provide better care to our patients.(mid)

… the best part was we were allowed to make mistakes and learn … (med)

### Bridging theory to practice

Both study groups expressed that the hands-on practice helped to tie in their theoretical knowledge with practical knowledge, supplementing whatever they had learnt in the lectures and pre-reading material with the actual clinical exposure required to conduct the examination. Students found that they were able to better visualise what they had learnt in classes, and their “understanding of concepts were enhanced”.

This workshop helped me a lot to understand the topic in theory as well as in practical knowledge. It’s a very good idea because we never get such opportunity to practice … (med)

Basic practical concepts made clearer. (mid)

It helps a lot to understand the theory portion through a practical point of view.(med)

This simulation workshop is totally different from clinical session or a tutorial as in this workshop, we learnt many things without opening books whereas in tutorial, it remains limited to books … (mid)

Perhaps the best way to demonstrate medical science to an undergrad student...to enhance their understanding...(med)

… Simulation workshop is very effective as compared to clinical session or tutorial. During tutorial we only get theoretical knowledge and during clinical sessions we do not get much opportunity. (mid)

### Valuing interprofessional interaction

Students felt that the interprofessional activity allowed them to coordinate and share their ideas and knowledge in a mutually beneficial manner. After the workshop, they expressed greater trust and understanding in one another and recognised that such training can improve the overall quality of care delivered to patients.

Interprofessional activity is very valuable, so that medical & midwifery personnel can work together as team, which can improve the quality of care, which we provide to their patients...(med)

Interprofessional activity is useful, as in future, during our working, we have to work together. We practiced coordination that will lead to a successful result. (mid)

Thank you for IPE. The interprofessional activity is more effective to work with each other because one person cannot do everything; we all depend on each other in clinical practice. We all are important in clinical practice...(mid)

This has been a very productive experience in the course of interprofessional acitivity. It was a really great way to learn material and for visualisation. Because the activty was performed with MBBS and nursing students, this provided us with a real time simulation for both of us. (med)

### Ensuring equal learning opportunity for all participating professional groups

Another prominent theme that appeared in the feedback revolved around the equality of opportunity offered to both sets of students and dominance of one group over another may occur in an IPE setting. While both groups of students were grateful for the opportunity to take part in the IPE, some midwifery students felt that the medical students were more dominant during the IPE and deprived them of the chance to participate equally in the procedures.

Interprofessional activity is a great concept to combine medical and midwifery as with this combination, higher levels of excellence can be achieved in health sector, but during the simulation workshop, medical students were dominant in performing procedures and midwifery students didn't get a fair chance. (mid)

In this interprofessional activity with medical and midwifery students there were more opportunities given to medical student as they were dominating during demonstration and doing more...(mid)

It is nice in the medical field to see the medical and nursing students to be seen as equal. And in this workshop or simulation we had that opportunity to share our ideas and knowledge with each other beside without comparison or bias. But then also medical student got a little bit of special consideration...(mid)

## Discussion

This paper explores the benefits of interprofessional simulation in an undergraduate low-resource healthcare setting. Both groups of students found the stations highly relevant to their course and felt more confident in their clinical examination skills after the workshop. They found the workshop beneficial and were inclined towards having more sessions in the future. SBE was identified to be different from other forms of learning, like didactic methods. This may be ascribed to the provision of hands-on practice being valuable as it tied the theoretical knowledge with practical skills. The ability to make mistakes without severe consequences and the removal of clinical time pressures was seen to enhance the learning process. The interprofessional workshop was identified as a new concept and was suggested to improve relationships between the two professions.

Engagement in learning is crucial and necessary for health professional education. Healthcare simulation can improve engagement by creating clinical situations that appeal to students’ interest, attention and learning. To optimise learner engagement, simulation needs to be physically, cognitively and emotionally engaging [16]. Through hands-on practice, learners can engage in constructive behaviour that builds greater confidence and understanding of the material. With the use of simulators, learners have full control over the material and synthesise information in a personally meaningful manner [17]. Additionally, this form of learning demands a higher level of cognitive functioning from learners compared to didactic teaching, as learners must physically manipulate objects, draw observations and piece information together [18]. This concept can be explained through socio-materiality [19], where humans interact with materials that play the role of being fundamental tools in their learning. Active learning sought by participants through these deliberate interactions can therefore lead to greater knowledge gains and positively influence long-term retention of information.

Simulation can help to reinforce clinical skills in a safe and educationally orientated environment. By eliminating the time pressure associated with a busy clinical setting, a stress-free environment is created for learners to experiment and learn at their own individual pace [20], as was demonstrated by our study. Patients treated by an individual or team during their learning phase are at higher risk of morbidity [21]. Students understand this risk and know that potential injury could be caused if the necessary technique, knowledge and skills have not been acquired [9]. Whilst this awareness may help them to recognise their scope and limitations of practice, it may occasionally lead to a state of anxiety, which may be detrimental to learning. Simulation allows learners to retain the ability to make mistakes (as highlighted here by the students) and learn the lessons that come with it, at no cost to patient safety.

In order to encourage interaction and maximise learning, the simulation must also behave in a realistic manner. In simulation, realism refers to how similar the simulation is related to reality. Highly realistic simulation can provide students with opportunities to engage in hands-on application of theoretical knowledge that supports the transfer of theoretical knowledge to practical skills while also improving critical thinking [22]. However, highly realistic simulators need not be expensive or use complex technology, in order to maximise cost-effectiveness in a low-income setting [23]. Effective use of part-task trainers can provide the realism required for students to engage in a meaningful learning activity, especially if the learning objectives are focussed on specific task-based learning (as in this study) either by individual participants or in a team environment. Creating effective learning scenarios which reflect the complexities of the real world can also lead to better learner engagement [21]. Not only can such simulations improve the teaching of procedural and technical aspects of healthcare but also through better engagement, it increase the learner’s ability to communicate and care for their patients [16].

While both groups of students appeared to be immersed in the activity, the results suggested an underlying perception of contest about learning opportunities. These perceptions may potentially weaken the development of collaborations and role-sharing, that IPE activities intend to foster. A sensitivity towards looking after learning needs and providing equal opportunities to each group may be necessary for creating a positive learning experience in an IPE setting [24]. This is particularly relevant towards creating interprofessional identities with complementary roles [25]. In low- and middle-income countries like India, a strong hierarchical system is still prominent in the healthcare sector [26]. A culture of medical dominance and nursing subservience is pervasive in many hospitals, where nurses have little input and no leadership in patient care [26, 27]. During the workshop, some of the nursing students expressed that medical students were more dominant in performing procedures, suggesting that the power differential is deeply instilled in the system even at undergraduate levels. The imbalance in this power relation can be partially attributed to traditional stereotypes of nurses being subordinates of doctors and the differences in prestige, education and respect associated with each profession [28].

In another study, a cross-sectional survey of midwifery and medical students revealed that both groups perceived their own role in the care of obstetric patients to be more important than that of the other group [29]. By failing to recognise the complementary nature of each group’s roles, a patient’s care could potentially be compromised. Given that certain components of the obstetrics and gynaecology curriculum for medical students mirror those in the midwifery course, IPE and sharing of teaching resources could build a culture of co-operative learning between students from both disciplines and improve future working relationships [30]. Early exposure to interprofessional education presents an opportunity for core clinical skills to be standardised amongst both faculties. By adopting similar learning outcomes and procedural skills, students from both faculties will have more confidence in their colleagues’ abilities, and this may translate to fewer differences of opinion when they eventually become doctors and midwives [24]. Ultimately, this can build mutual appreciation between the team and reduce potential sources of conflict in patient management [29].

There is a vast difference between the roles of doctors and nurses. One cannot function without the other, and it is crucial to bridge the gap between the two professions. Professional differences stand in the way of optimal patient care. They create preconceived negative stereotypes that can be passed down from senior staff members to juniors in the workplace and lead to significant confusion over the roles and responsibilities of each group [31]. Interprofessional education offers an opportunity to depart from this out-dated, hierarchical, towards a more inclusive and holistic healthcare system. Developing a supportive environment of psychological safety between the two interprofessional teams, where individuals feel comfortable participating in discussion and raising opinions without fear of being judged, criticised or ridiculed, will be essential. This can lead to the sharing of power between doctors and nurses as individuals become more assertive in discussions, participate more in shared decision-making and take on more responsibilities in patient care [32]. The inclusion of these interprofessional competencies has been suggested to make interprofessional activities more effective for the curriculum [33]. In the specific context of interprofessional simulation, guidelines have been published by the International Nursing Association for Clinical Simulation and Learning (INACSL) [34]. It highlights issues like recognition of barriers to implementation, such as funding problems, low resources and poor institutional support.

The study has the limitation of being a single intervention study in an isolated medical and midwifery student cohort. There is a possibility that the results may be affected if performed multiple times, or in different sets of learners in another setting. The study is also based on self-reported learning and confidence acquired by students, with data collection performed immediately after the intervention. It may be interesting to evaluate whether the skills were learnt and if they could be performed with accuracy either in the simulated setting or in clinical practice. However, this was currently considered to be beyond the scope of this work and is being considered for future research.

## Conclusions

As simulation-based learning in the field of obstetrics and gynaecology is gradually gaining prominence in the developed world, an understanding of how best to engage learners with it and maximise learner outcomes is crucial. By capitalising on the strengths of simulation-based learning that was identified in the article, we can deliver more effective teaching to midwifery and medical students specially, in the setting of low-middle-income countries, where interprofessional or simulation programs are scarce. Additionally, through the inclusion of training at an undergraduate level, we can improve communication and collaboration between doctors and midwives and break down professional boundaries before they form. This is particularly relevant for low–middle-income countries with high rates of maternal and perinatal mortality and presence of a deep-rooted hierarchical system in healthcare. More studies are required in these settings to evaluate the impact on team behaviour and outcomes for better understanding of the influence, such initiatives can make towards an improved patient care.

## Abbreviations

IPE: Interprofessional education
SBE: Simulation based education
WHIPLS: Women’s Health Interprofessional Learning by Simulation
